# Supplementary material for: Point mutations of the mitochondrial chaperone TRAP1 affect its functions and pro-neoplastic activity
Source: Cell Death Dis. 2025 Mar 12;16(1):172. doi: 10.1038/s41419-025-07467-6 (PMC11903959; doi:10.1038/s41419-025-07467-6)
Supplement: Supplementary file 1 — Supplementary Fig. legends [file 41419_2025_7467_MOESM1_ESM.pdf]

Supplementary Fig 1. **High throughput screening and classification of TRAP1 point mutations**. **A** Box plot of human TRAP1 (ENSG00000126602.10) gene expression data from GTEx Portal. Expression values are expressed as logarithm of transcripts per million (TPM). Boxplots are expressed as median and 25th and 75th percentiles. **B** Comparison between TRAP1 expression in cancer and healthy samples. Red boxplots are for cancer sample, while blue boxplots are for healthy tissue. Expression data calculated using the Affymetrix HG-U133Plus2 dataset (GPL570) as reported in the GENT2 database. In **A** and **B**, dots are for outliers (1.5 times above or below the interquartile range). **C** Classification of 310 TRAP1 variants retrieved on MAVISPs according to their effect on protein stability in neutral (66%), uncertain (25.5%) and destabilizing (8.5) ones.

Supplementary Figure 2. Effect of point mutations on TRAP1 stability. A Table indicating the position of each mutation in both human and Zebrafish TRAP1 (hTRAP1 and zTRAP1). In zTRAP1, protein residue numbering is offset by 15 residues relative to the human protein. **B** Occupancy of H-bond interactions involving residue 615 of zTRAP1 (600 in hTrap1) in protomer A or B in wild-type and T615P zTrap1 during MD trajectories (12000 frames, 600ns). Numbering of residues refers to zTrap1 sequence as in PDB code 4IPE. **C** Pairwise Cα Distance Fluctuation (DF) matrices of WT and mutant hTRAP1-T600P together with the percentage difference matrix %ΔDF. In DF matrices DF scores are coloured according to a grey scale as indicated in the colour bars. In the $\%\Delta DF_{mut}$ blue areas (positive values) correspond to lower mechanical coordination in Trap1 mutant than in the WT protein whereas orange ones (negative values) indicate a higher coordination in the first. Grey/white areas are those unaffected by the mutation. On the x axis, numbering is as in PDB code 4IPE and the values indicated correspond to the domain division. For each protomer residue numbering is from 85 to 719 and the assignment of domains is as follows: N-terminal Domain (NTD) residues 85-310; Middle Domain (MD), divided in the subdomains Large Middle Domain (LMD), residues 311-470 and Small Middle Domain (SMD) residues 471-586; C-Terminal Domain (CTD) residues 587-719. **D** Western-blot showing TRAP1 protein levels in sMPNST cells expressing the native protein (scramble) or re-expressing either the wild-type or the reported mutant forms of human TRAP1 after knocking-out the endogenous protein (TRAP1 KO). Citrate synthase was used as a loading control.

Supplementary Figure 3. Effect of point mutations on TRAP1 molecular dynamics. A Occupancy of H-bond interactions involving residue 275 (260 in hTrap1) in protomer A (light blue) or B (dark yellow) in WT and D260N zTrap1 during MD trajectories (12000 frames, 600ns). **B** Structure of WT zTrap1 with P396 (P381 for hTrap1) and interacting residues listed in table shown as sticks together with ATP. In **A** and **B**, numbering is relative to zTrap1 sequence as in PDB code 4IPE. To convert to hTrap1 numbering 15 should be subtracted. **C** Mechanical connectivity index for each mutant along the sequence (η_{mut}). On the x axis numbering of zTrap1 as in PDB code 4IPE is shown together with the corresponding domains of prot. A and B. For each protomer residue numbering is from 85 to 719 and the division in domains is as follows: N-terminal Domain (NTD) residues 85-310; Middle Domain (MD), divided in the subdomains Large Middle Domain (LMD), residues 311-470 and Small Middle Domain (SMD) residues 471-586; C-Terminal Domain (CTD) residues 587-719.

Supplementary Figure 4. TRAP1 point mutations differentially affect SDH activity and mitochondrial bioenergetics. A Succinate dehydrogenase (SDH) activity measured in sMPNST scr or TRAP1 KO cells, and in KO cells re-expressing human WT TRAP1. Data are

reported as mean±S.E.M. of 3 independent experiments and analyzed with a two-tail unpaired Student's *t* test with each mutant compared to TRAP1 KO cells (**, p value < 0,01; ****, p value < 0,0001; n.s., non-significant). **B** Western blot assessing the protein level of the subunit A of SDH in sMPNST cells expressing either the WT or mutant forms of TRAP1. Citrate synthase was used as a loading control. **C** Reproduction of PDB code 7KCM showing TRAP1 bound to the model client protein SDHB (in black). Valine 556 is shown in stick and a zoomed view of its contact with SDHB is shown in the inset. **D** Representative OCR traces measured in sMPNST cells expressing either human wild-type or mutant forms of TRAP1. Subsequent additions of oligomycin (1 µM/ml), which allows determination of respiration coupled to ATP synthesis, of the proton uncoupler carbonyl cyanide-4-(trifluoromethoxy)-phenylhydrazone (FCCP, 400 nM), which indicates the maximal and the spare respiratory capacity, and of the respiratory complex I and III inhibitors rotenone (1 µM) and antimycin A (1 µM), respectively, were carried out as indicated. **E** energy map for sMPNST cells expressing either the wild-type or P381S variant of human TRAP1. Raw data were analyzed using Seahorse Wave Software (version 2.3.0.19) prior to graphical presentation using Graphpad Prism. Data are reported as mean ± SD values (n=3); asterisks indicate significant differences (**: $p < 0.01$, *: $p < 0.05$; Student's *t* test analysis).

Supplementary Figure 5. Effect of TRAP1 point mutations on its pro-neoplastic activity. **A** Representative images of foci formed by sMPNST cells. **B-G** Focus-forming assay in SCR and TRAP1 KO sMPNST cells (**B, C**) and in KO cells re-expressing h-TRAP1-WT or mutant forms (**D-G**). Cells were grown for 10 days with or without the selective TRAP1 inhibitor compound 5 (25 µM). Foci were quantified using an integrated density parameter that evaluates both their surface and thickness by ImageJ Software. Data are presented as mean ±SEM of at least 3 independent experiments with 3 replicates for each one; ***: $p < 0.001$ with one-way ANOVA with Bonferroni's test. **H** Summary table illustrating the effects of TRAP1 mutations on protein activity, as well as on the bioenergetic and tumorigenic properties of sMPNST cells. Changes in the reported properties are relative to hTRAP1-WT expressing cells.
