# Supplementary figures and images for: Point mutations of the mitochondrial chaperone TRAP1 affect its functions and pro-neoplastic activity
Source: Cell Death Dis. 2025 Mar 12;16(1):172. doi: 10.1038/s41419-025-07467-6 (PMC11903959; doi:10.1038/s41419-025-07467-6)

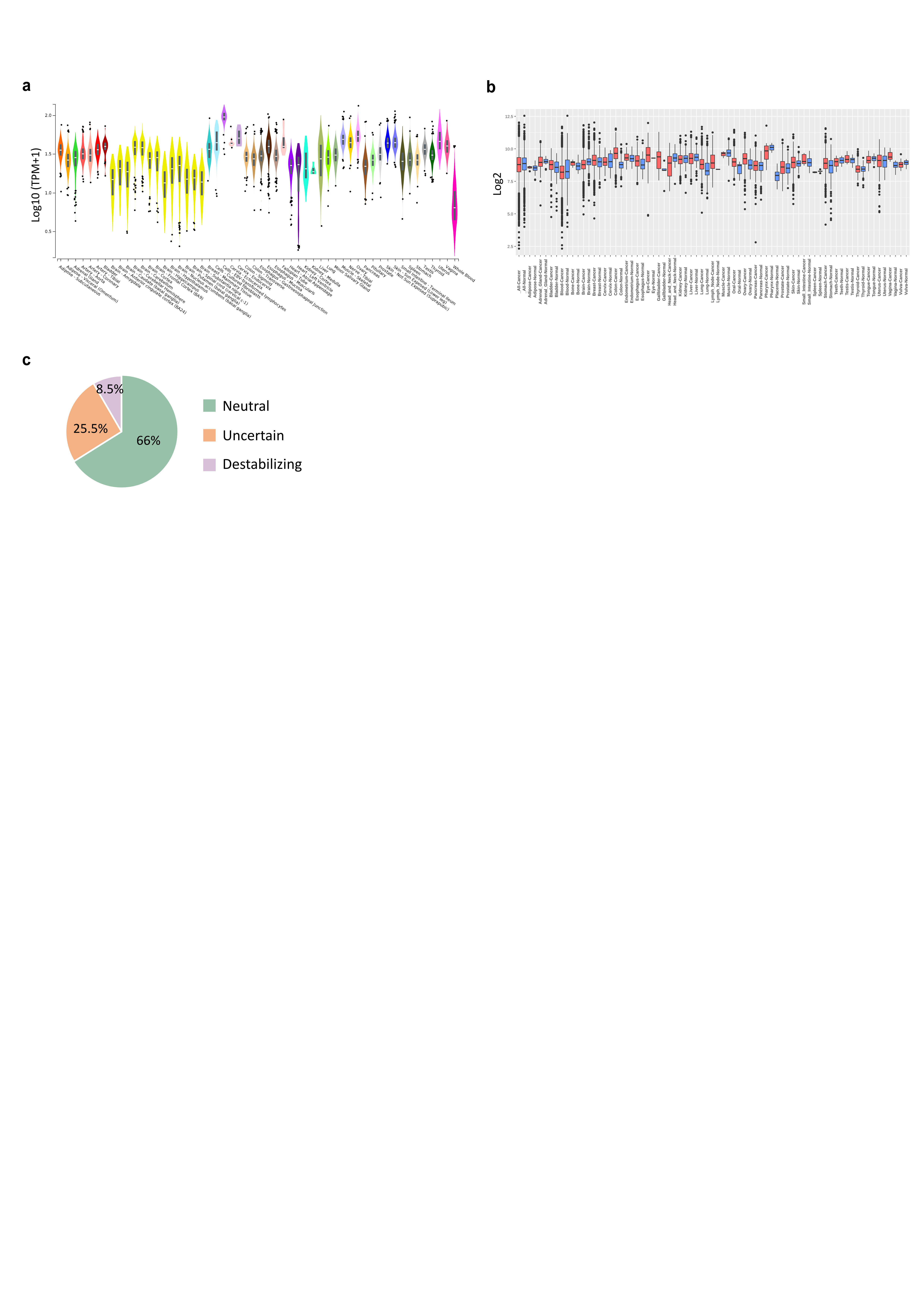

Supplement: Supplementary file 2 — Supplementary Figure 1 [file 41419_2025_7467_MOESM2_ESM.png]

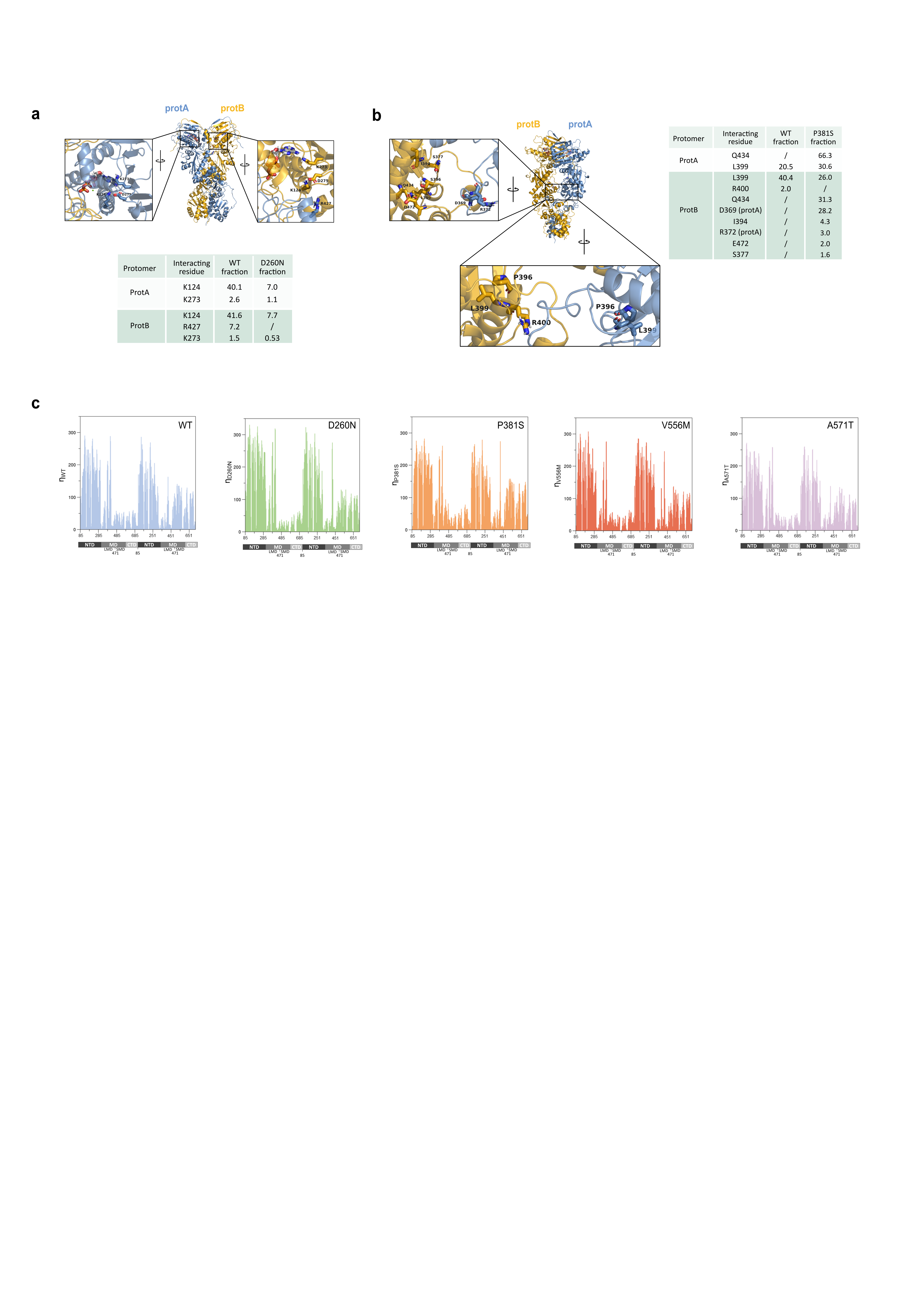

Supplement: Supplementary file 4 — Supplementary Figure 3 [file 41419_2025_7467_MOESM4_ESM.png]

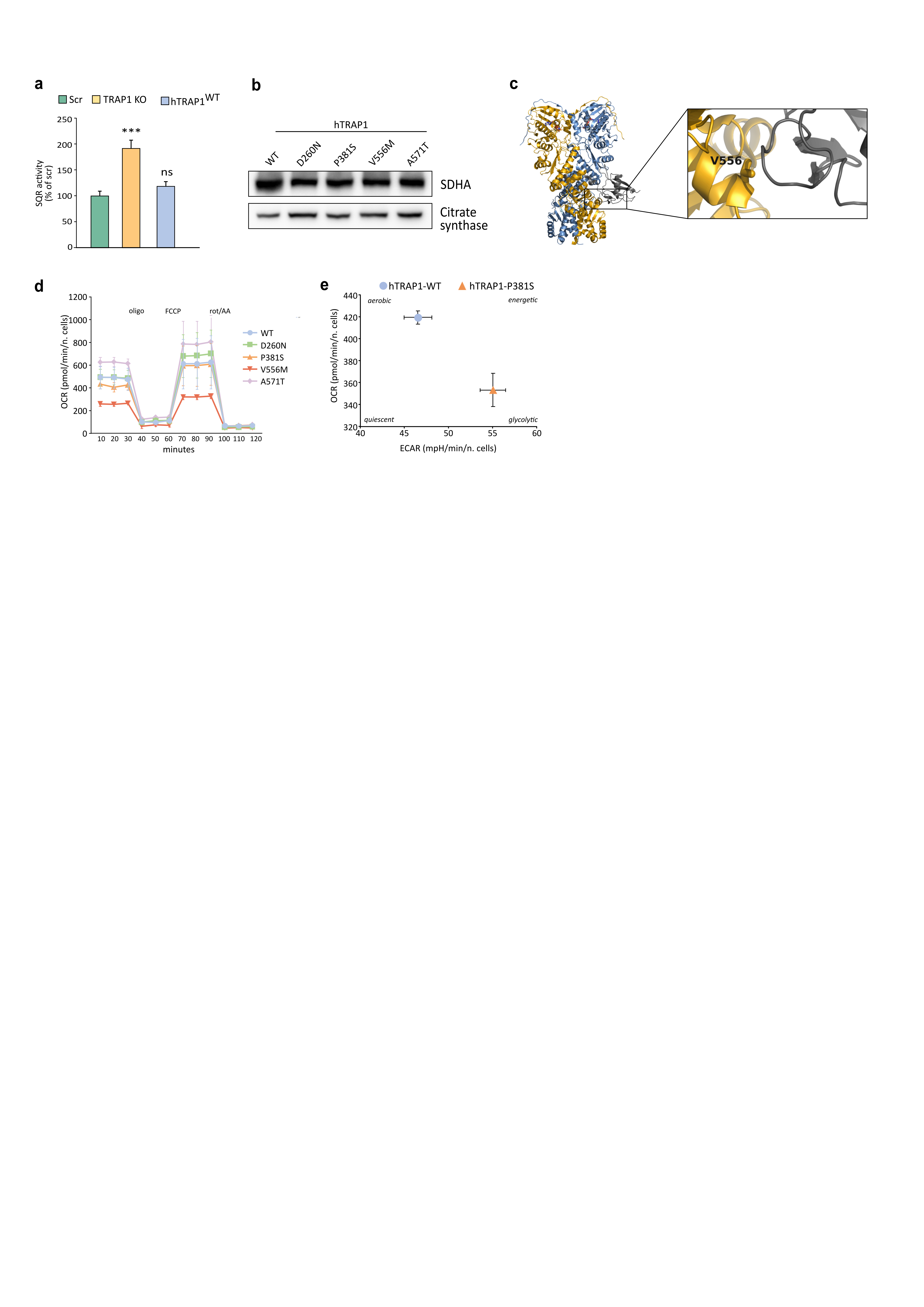

Supplement: Supplementary file 5 — Supplementary Figure 4 [file 41419_2025_7467_MOESM5_ESM.png]

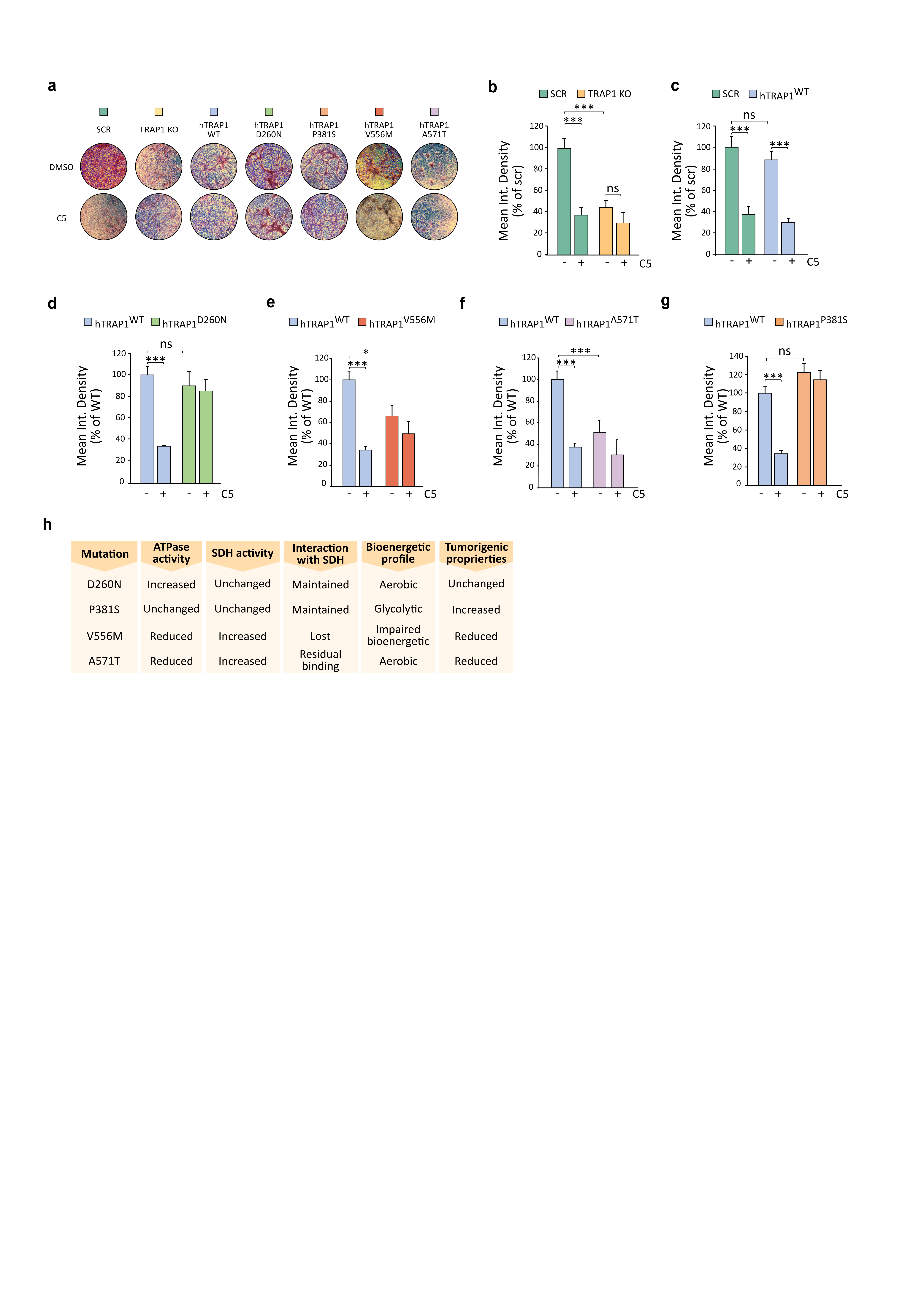

Supplement: Supplementary file 6 — Supplementary Figure 5 [file 41419_2025_7467_MOESM6_ESM.png]

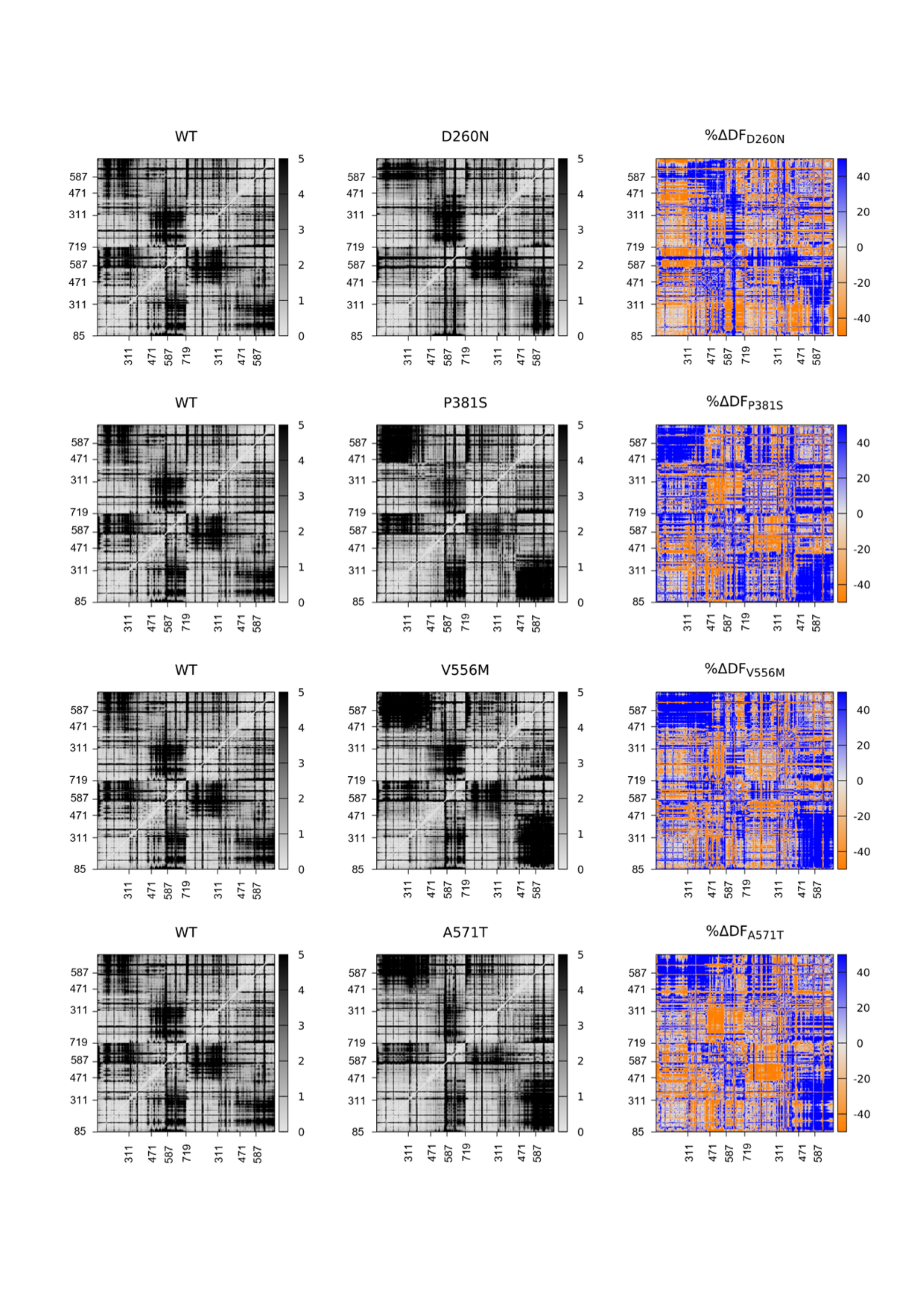

Supplement: Supplementary file 8 — Additional file 2 [file 41419_2025_7467_MOESM8_ESM.png]

Figure 2d

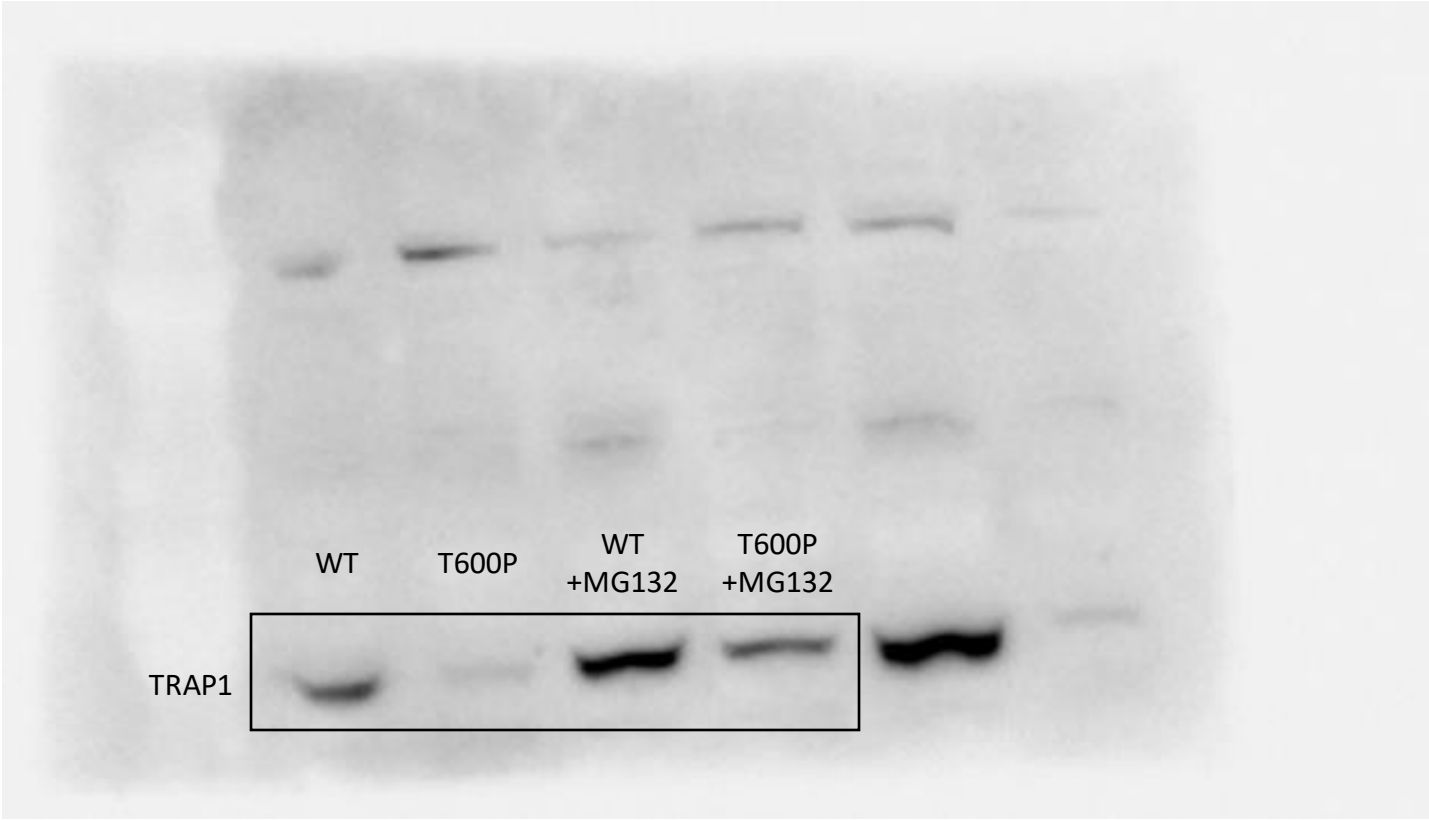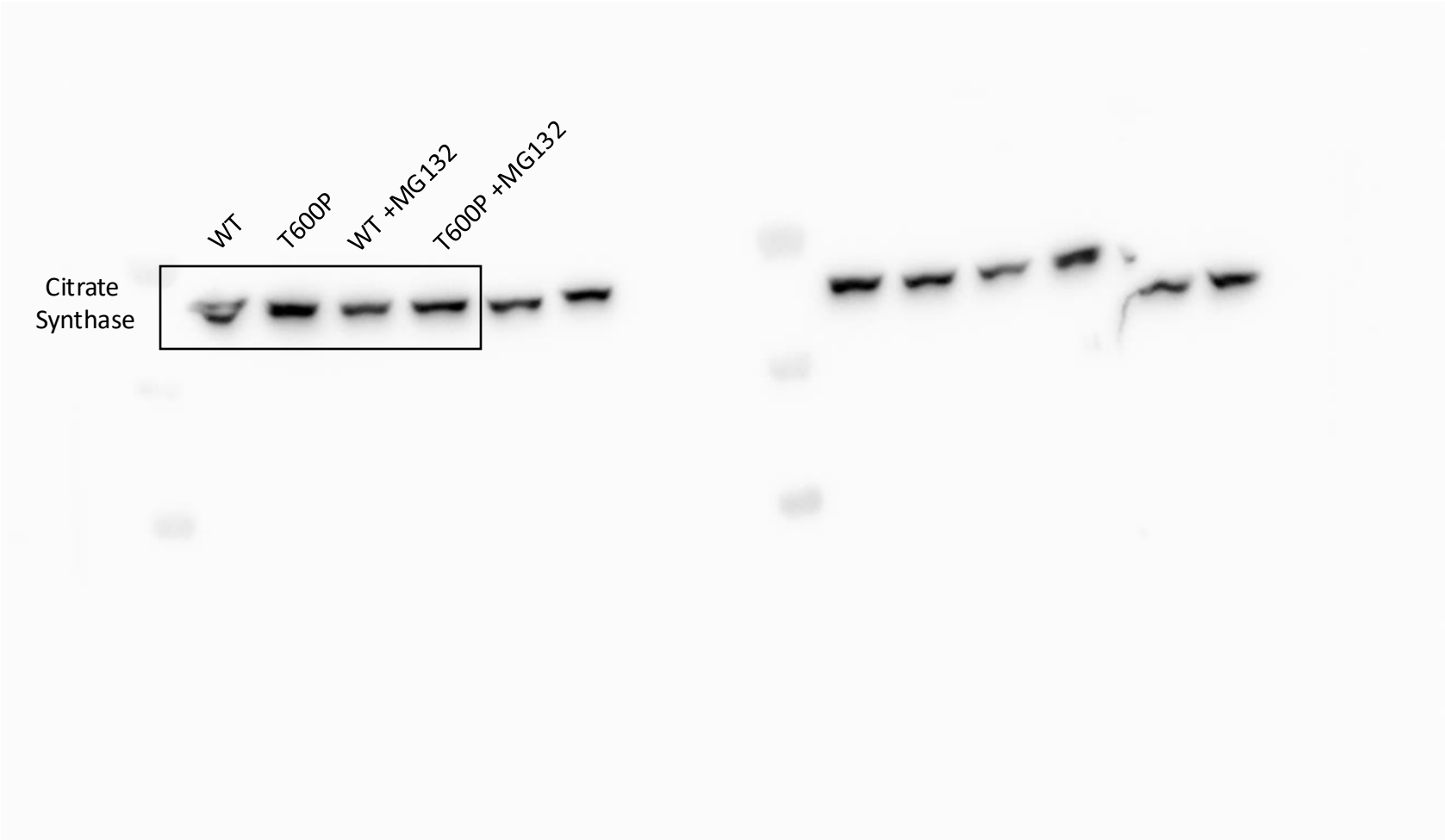

Figure 2e

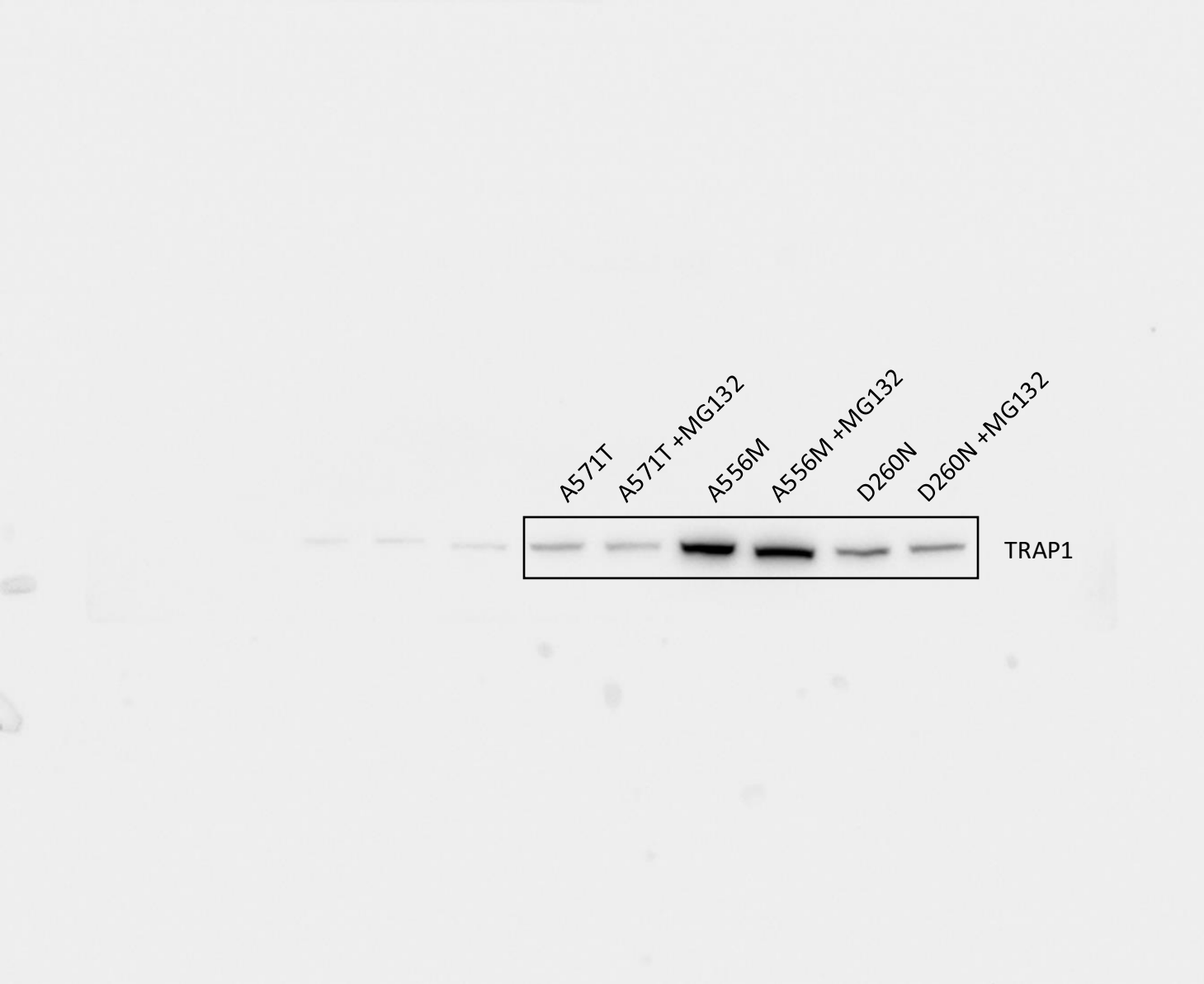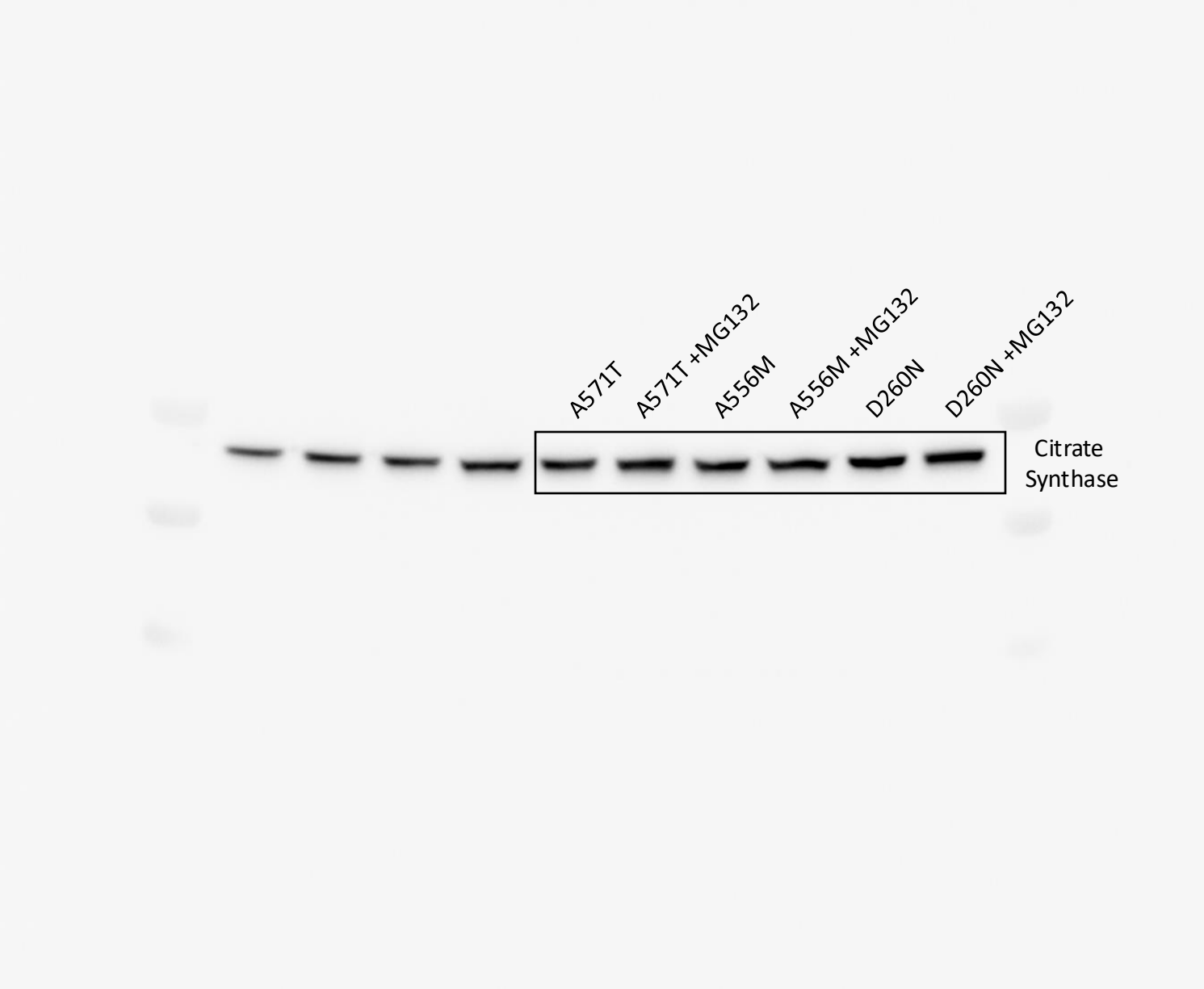

Supplementary Figure 2d

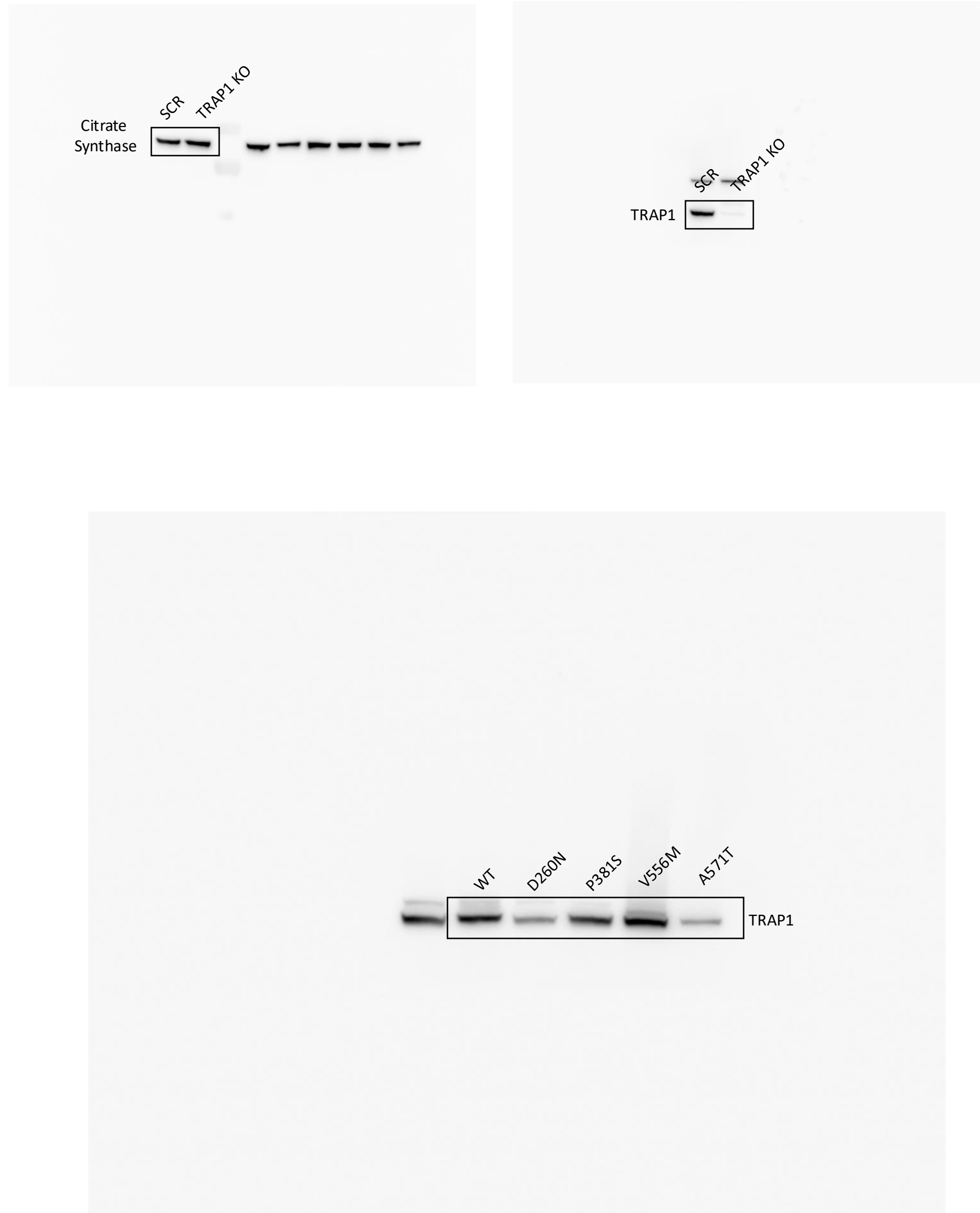

Figure 2e

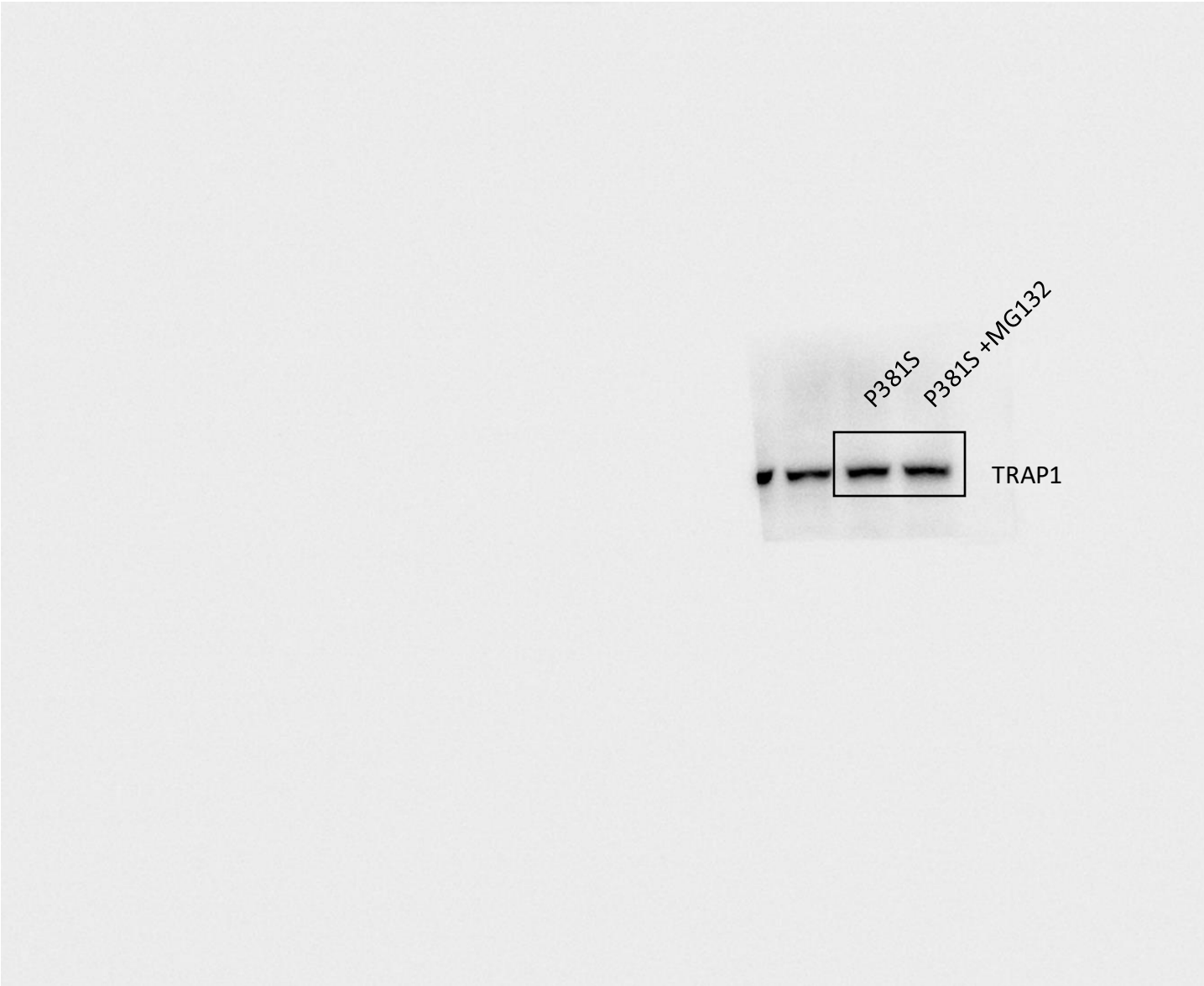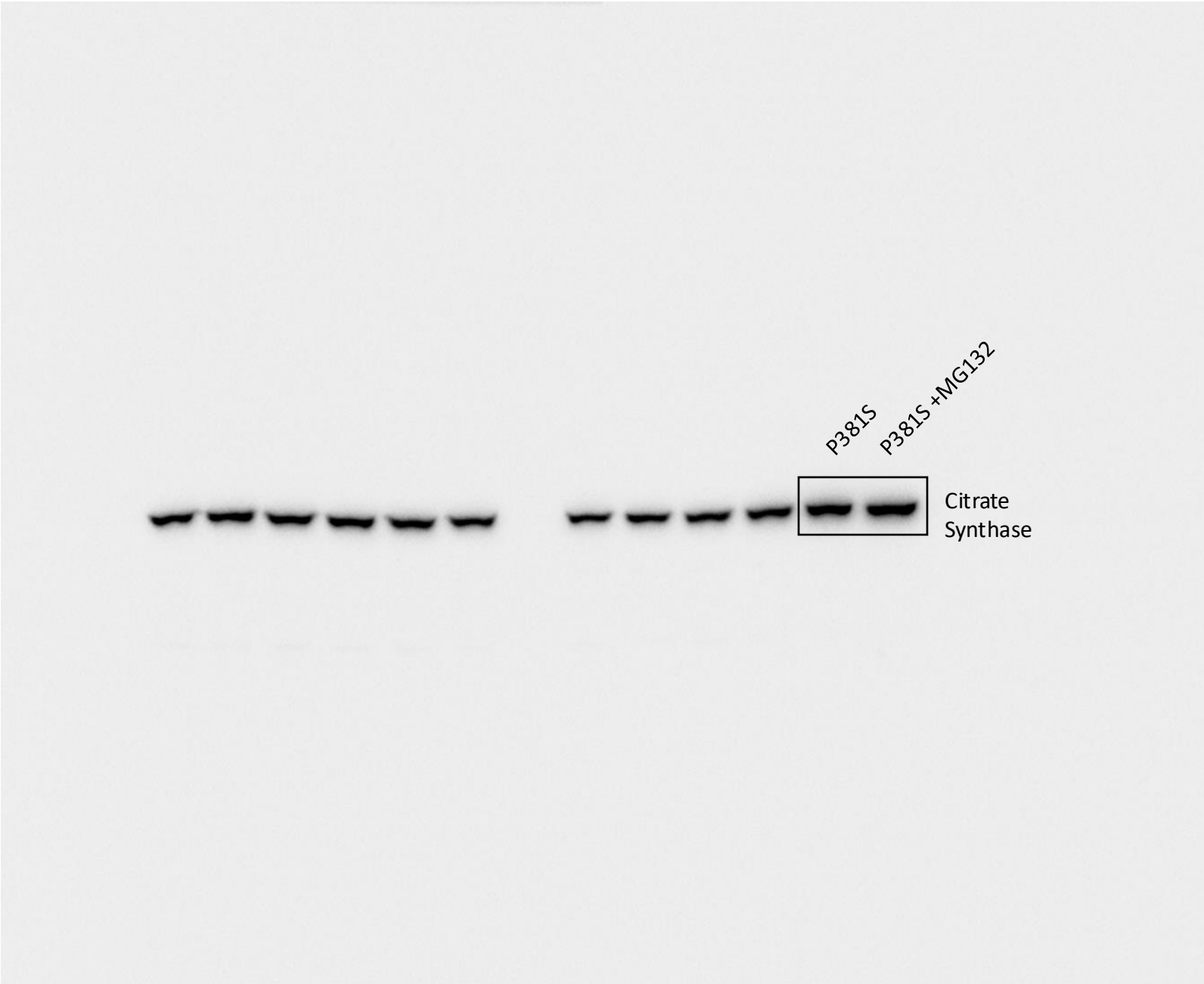

Figure 4c

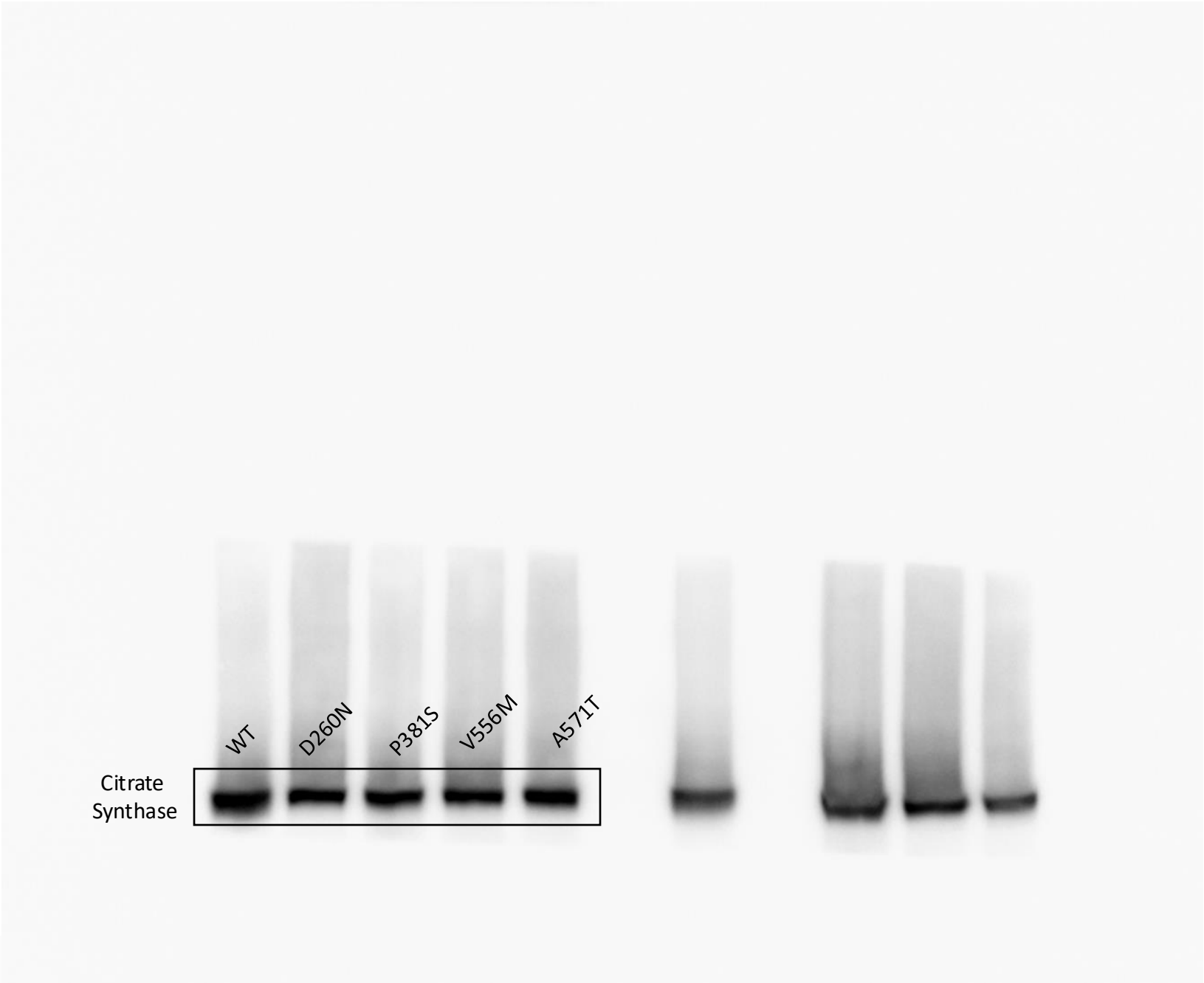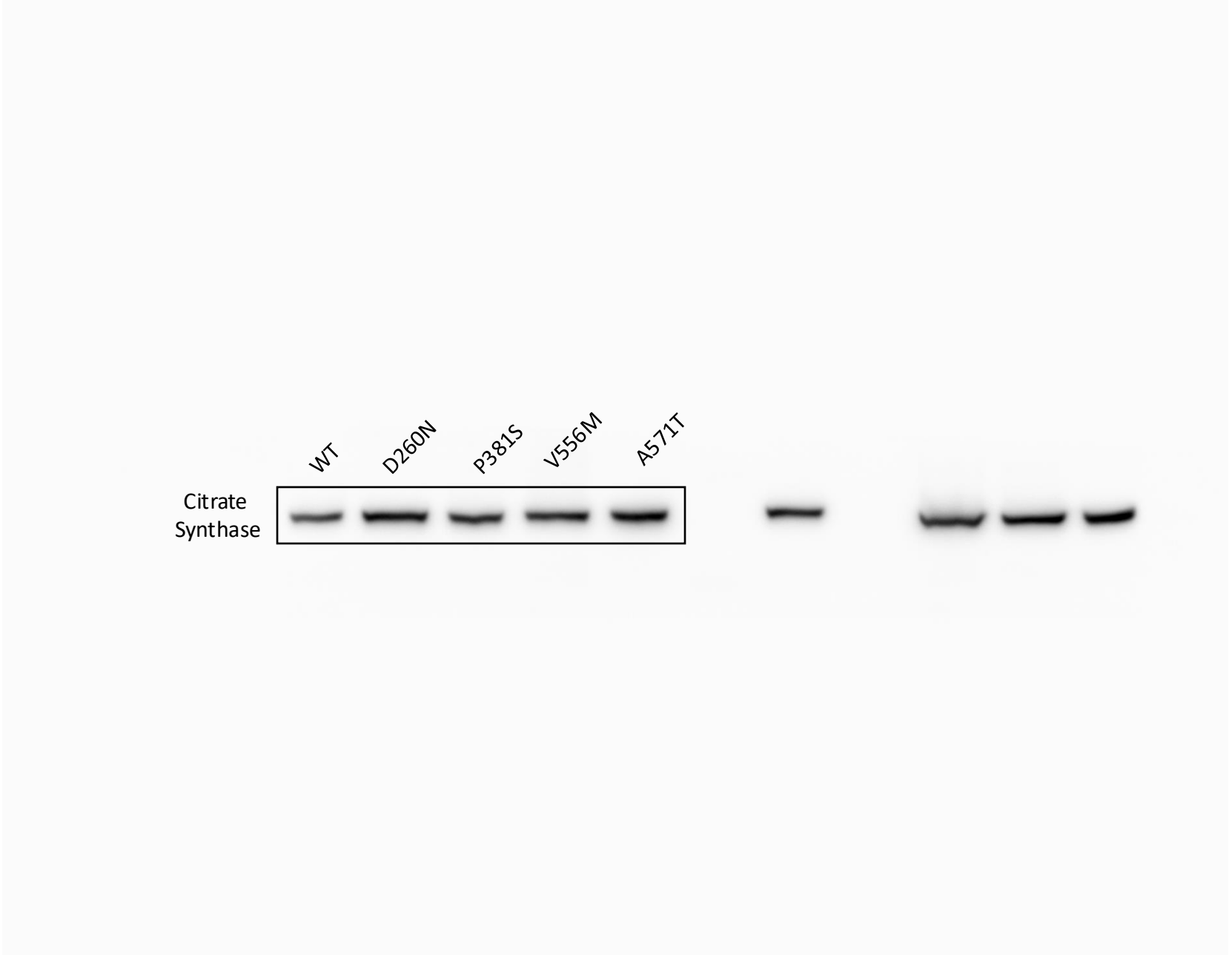

Figure 4b

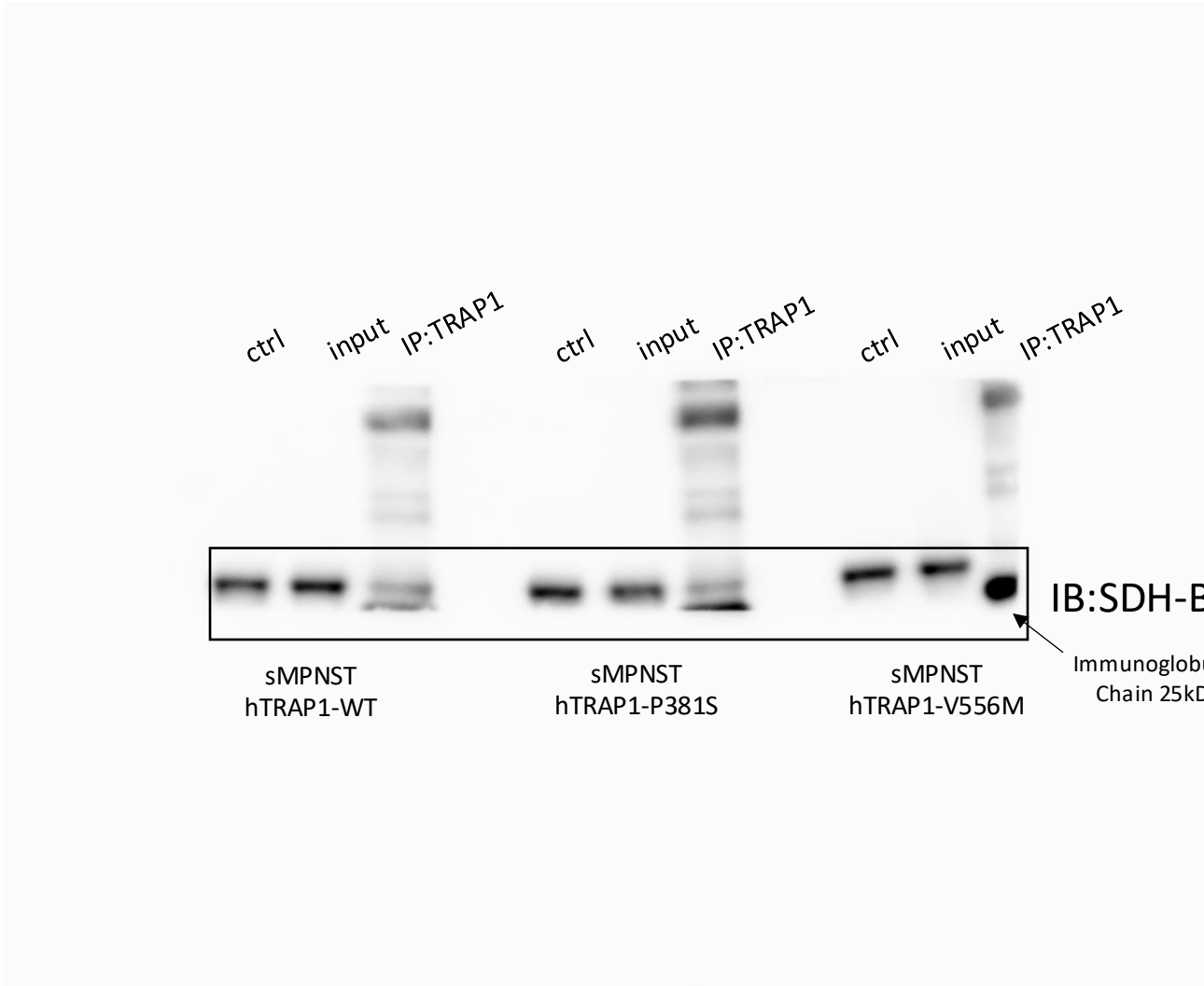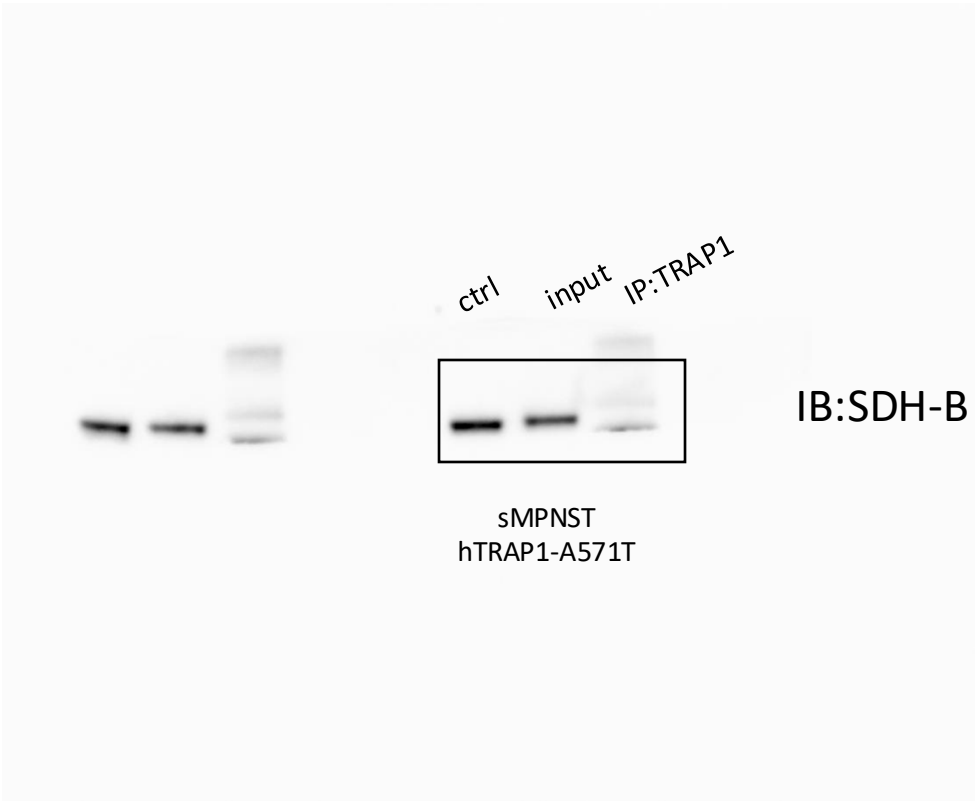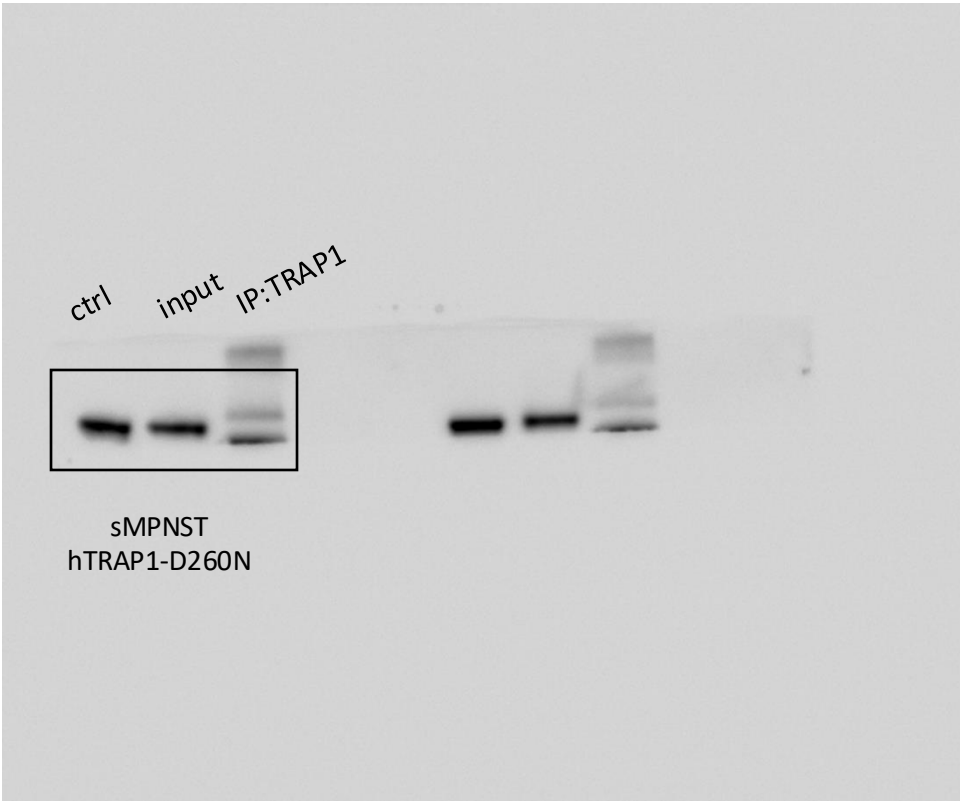

Figure 4b

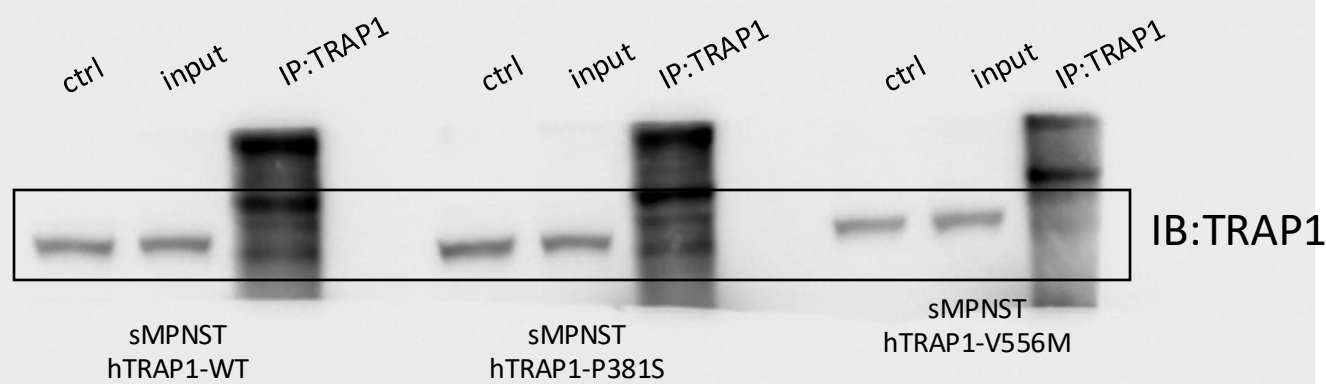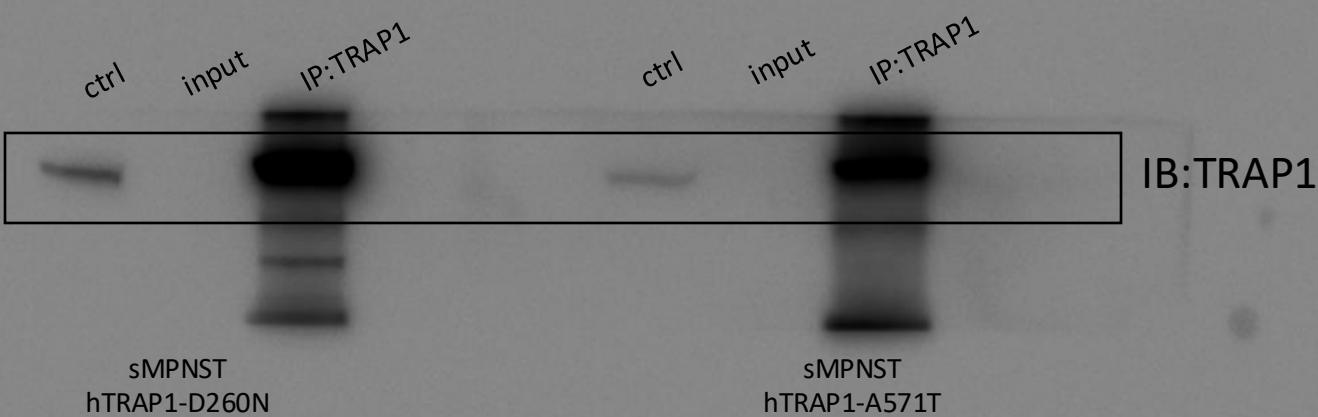

Supplement: Supplementary file 9 — Original data files [file 41419_2025_7467_MOESM9_ESM.pdf]
